# Recent Advances in the Synthesis and Application of Tellurium Semiconductors

**DOI:** 10.3390/nano16120725

**Published:** 2026-06-11

**Authors:** Hao Yang, Zhiyi Lyu, Hoo-Jeong Lee

**Affiliations:** 1 School of Advanced Materials Science and Engineering, Sungkyunkwan University, Suwon 16419, Republic of Korea; yanghao0393@g.skku.edu; 2 Department of Biophysics, Sungkyunkwan University, Suwon 16419, Republic of Korea; 3 College of Engineering, Eastern Institute of Technology, Ningbo 315200, China; 4 Department of Smart Fab. Technology, Sungkyunkwan University, Suwon 16419, Republic of Korea

**Keywords:** tellurium nanowire, tellurene, field-effect transistors, logic-gate, photodetectors, memristor, artificial synapse

## Abstract

Tellurium (Te), an attractive p-type van der Waals semiconductor, has been considered a promising candidate in electrical applications due to its unique one-dimensional chiral atomic-helical-chain structure, tunable bandgap, and ultrahigh hole mobility. This review summarizes recent advances in the controlled synthesis of Te semiconductor nanostructures, including one-dimensional tellurium nanowires and two-dimensional tellurene in the form of nanosheets and thin films. We further highlight emerging electrical applications of Te in field-effect transistors, logic circuits, photodetectors, memristors, and artificial synapse devices. Finally, current challenges and future opportunities for the commercialization of Te-based electronic and optoelectronic devices, particularly for neuromorphic and in-sensor computing systems, are discussed.

## 1. Introduction

Tellurium belongs to the Group VI chalcogen family. Both bulk tellurium crystals and their nanostructures, including nanobelts, nanotubes, nanowires, and nanosheets, adopt a trigonal crystal structure [1,2,3,4,5,6]. This structure is composed of one-dimensional helical Te chains that are packed together through van der Waals interactions [7,8,9,10]. Within an individual chain, each Te atom connects with neighboring atoms via two directional covalent bonds, which facilitates the characteristic helical-chain arrangement [11]. The coexistence of strong intrachain covalent bonding and weak interchain van der Waals coupling endows tellurium with pronounced structural and electronic anisotropy. As a representative p-type semiconductor, Te nanostructures, including one-dimensional tellurium nanowires (TeNWs) and two-dimensional tellurene, have attracted increasing interest for diverse electronic and optoelectronic applications because of their tunable bandgap (0.35–1.04 eV) and excellent carrier-transport properties [12,13,14,15,16,17,18].

Owing to their unique structures and attractive properties, a variety of synthetic strategies have been developed to prepare high-quality tellurium nanostructures, including hydrothermal synthesis, chemical vapor deposition (CVD), physical vapor deposition (PVD), ultrasound-assisted synthesis, thermal evaporation, liquid-phase exfoliation, molecular beam epitaxy (MBE), and magnetron sputtering [12,19,20,21,22,23,24,25,26,27,28,29,30]. In addition, due to their unique crystal structure, techniques such as Langmuir–Blodgett (LB) assembly, bar coating, and layer-by-layer assembly have been used to transform 1D TeNWs into 2D films [31,32,33,34]. These strategies have already been explored in advanced electronics, optoelectronics, and large-scale integrated circuits [17,35,36,37,38,39,40,41,42,43,44,45].

This review summarizes the synthesis strategies of TeNWs and tellurene, and recent advances in their emerging applications. Here, we specifically discuss their use in electronic and optoelectronic applications, including field-effect transistors, logic circuits, photodetectors, memristors, and artificial synapse devices. We also assess the current limitations of tellurium semiconductors and outline future directions for their fundamental study and practical applications.

## 2. Structure and Properties

**Figure 1:**
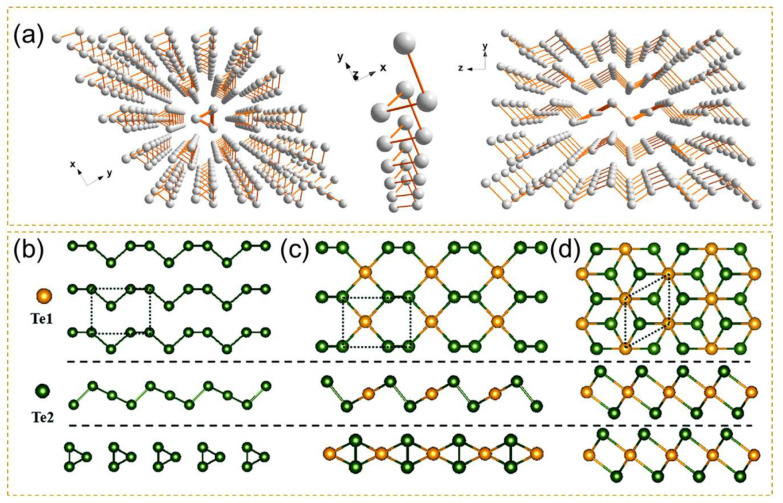
(**a**) Schematic illustration of the trigonal Te crystal structure viewed along the z-axis, in a single-molecule chain, and viewed from the x-axis. Adapted with permission from [21]. (**b**–**d**) Top and cross-section views of atomic structures of (**b**) α-Te, (**c**) β-Te and (**d**) γ-Te phases. Projections of each structure oriented along the xy-, xz- and yz-planes are shown from top to bottom, respectively. Te1 (orange) and Te2 (green) atoms are distinguished by color, respectively. Dashed lines indicate the optimized unit cell for each structure. Adapted with permission from [46].

In this section, we will discuss the crystal structure of Te. The crystal structure of Te consists of helical chains of Te atoms arranged in a trigonal configuration and stacked into a hexagonal array through van der Waals interactions. Figure 1a presents the Te crystal structure from different perspectives: viewed along the z-axis, as an individual molecular chain, and viewed along the x-axis, respectively [21].

This unique atomic configuration endows tellurium with intrinsic chirality and significant structural anisotropy. There are three representative phases: α-Te, β-Te, and γ-Te (Figure 1b–d) [46,47]. Among them, α-Te exhibits a three-coordinated structure, β-Te contains both three- and four-coordinated motifs, and γ-Te is a six-coordinated structure, demonstrating the diverse bonding configurations accessible to tellurium. Zhu et al. [48] examined 73 Te nanostructures by a first-principles study, including ribbon and nanowire configurations containing up to 37 helices per unit cell. Their calculations showed that α-Te and β-Te possess relatively high cohesive energies (2.62 and 2.56 eV per atom, respectively) and remain stable at room temperature. Conversely, γ-Te exhibits a lower cohesive energy of 2.46 eV per atom, leading to structural instability above 200 K. Furthermore, Qiao et al. [49] demonstrated that α-phase Te consists of 1D helical chains organized in a parallel arrangement with three Te atoms in each repeating unit, and that reducing the thickness to the monolayer limit can induce the formation of the β phase. Owing to this intrinsic anisotropy, few-layer Te exhibits several attractive properties, including high room-temperature mobility, a direct bandgap in the monolayer limit, and a layer-dependent band structure [50].

## 3. Synthesis of Tellurium Nanostructures

Owing to its unique structure and novel properties, the synthesis strategies of tellurium nanostructures have been extensively investigated. Various approaches have been developed for preparing one-dimensional TeNWs and two-dimensional tellurene. In this section, we focus on some representative synthesis techniques, including hydrothermal synthesis, thermal evaporation (TE), chemical vapor deposition (CVD), physical vapor deposition (PVD), and liquid-phase exfoliation (LPE).

### 3.1. Synthesis of Tellurium Nanowires

**Figure 2:**
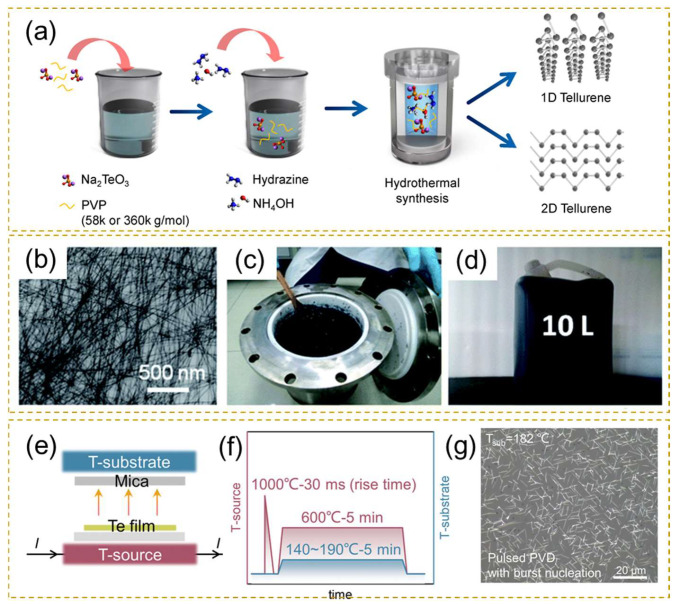
(**a**) Schematic diagram of hydrothermal synthesis of TeNWs and tellurene, adapted with permission from [61]. (**b**) SEM image of the as-prepared TeNWs; (**c**) photograph of high-yield TeNW production in a 16 L Teflon vessel; (**d**) photograph of TeNWs stored in a 10 L drum container, adapted with permission from [64]. (**e**) Schematic process of the PVD method; (**f**) designed temperature profile of the source and substrate for TeNW growth; (**g**) the optical image of the pulsed PVD with burst nucleation, adapted with permission from [65].

Hydrothermal synthesis is one of the most important routes for preparing TeNWs [51,52,53,54,55,56,57,58,59,60]. In a typical process, Te precursors are reduced in the presence of surfactants such as poly(vinyl pyrrolidone) (PVP) or cetyltrimethylammonium bromide (CTAB). Figure 2a illustrates a representative hydrothermal synthesis process [60,61]. In 2003, Qian et al. [51] reported a hydrothermal reduction strategy for the controlled synthesis of one-dimensional Te nanostructures, obtaining uniform nanowires with diameters of about 25 nm in alkaline solution containing PVP. The same group later developed a green chemical route to produce ultrathin TeNWs at a relatively low temperature of 90 °C, yielding nanowires with average diameters of about 7 nm and lengths of tens of micrometers. In this process, ascorbic acid served as the reductant, whereas CTAB acted as both a surfactant and a structure-directing agent [62]. The growth mechanism was described as a surfactant-assisted solid–solution–solid process. Initially, H^+^ ions from the ascorbic acid react with the Te precursor to form a white precipitate. Then, upon heating to 90 °C, the solid gradually dissolves and the weak reducing power of ascorbic acid slowly converts dissolved Te^4+^ species into Te atoms. CTAB then forms rod-like micelles that act as soft templates, directing the Te atoms to grow into long, ultrathin nanowires [19]. In 2006, the Yu et al. [63] used a PVP-assisted hydrothermal process to synthesize uniform single-crystalline TeNWs with diameters of 7–9 nm and lengths of several tens of micrometers; a representative TEM image is shown in Figure 2b. Hydrazine hydrate was used as the reducing agent, and the resulting ultrathin nanowires could be readily dispersed in water or ethanol. This method was later extended to sub-kilogram-scale production, as shown in Figure 2c,d, where as much as 150 g of ultrathin TeNWs was prepared in a 16 L Teflon vessel and stored in a 10 L plastic drum [64].

Although hydrothermal methods enable large-scale production of TeNWs, this method often suffers from relatively low crystallinity. To address this limitation, PVD has been used to grow TeNWs with improved crystal quality. Li et al. [65] reported a pulsed-PVD technique in which a Te thin film served as both the vapor source and a Joule heater, allowing precise control over the evaporation temperature and heating duration. As depicted in Figure 2e,f, this strategy produced uniform, high-density nanowires with high aspect ratios (thickness < 10 nm and length > 10 μm). The synthesized nanowires reached an average length of about 6 μm after 5 min of growth (Figure 2g). However, the TeNWs obtained by this method still exhibited largely random orientations, which limits device uniformity on a large scale. To overcome this issue, Wei et al. [66] developed a nanoscale groove-induced unidirectional epitaxial growth strategy for TeNWs using PVD. After annealing at 1300 °C, nanoscale grooves formed along the m-plane sapphire surface and guided the Te growth from random nucleation to nearly 95% unidirectional alignment. The controlled growth of oriented TeNWs is highly promising for large-area electronic and optoelectronic devices. More recently, He et al. [67] developed a molecular-engineering-based substrate manipulation strategy. By introducing specific molecules as an “anchor-rope” template onto an m-plane sapphire substrate with nanoscale grooves, they achieved wafer-scale growth of uniaxially aligned TeNW thin films, and statistical analysis showed highly ordered alignment with less than 5% angular deviation over a 1.3-inch scale. This molecule-engineered approach provides valuable insight into the controllable growth of low-dimensional Te materials.

### 3.2. Synthesis of Tellurene

Owing to its excellent electrical properties, two-dimensional tellurene exhibits remarkably high carrier mobility and significant structural anisotropy. In this section, we summarize some representative recent strategies for synthesizing tellurene.

**Figure 3:**
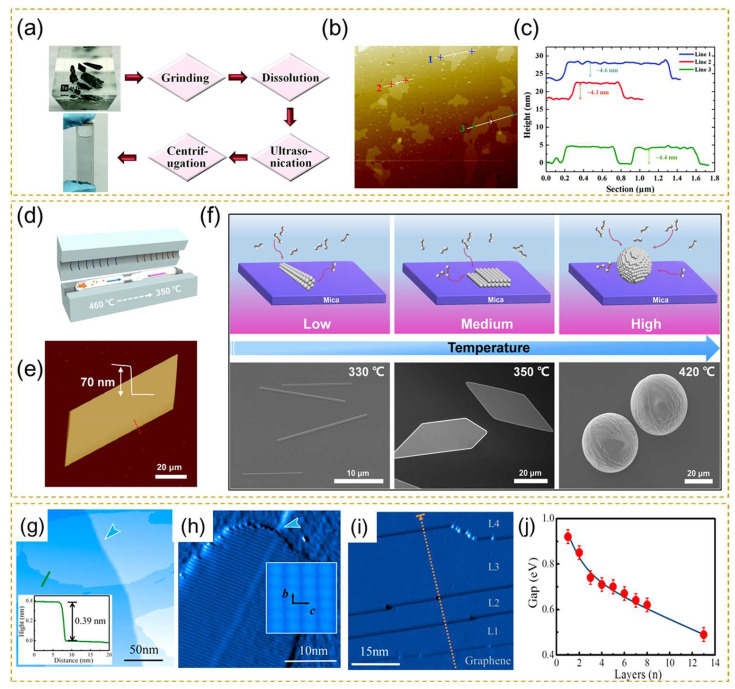
(**a**) Liquid phase exfoliation process of the tellurene dispersion; (**b**) AFM result of tellurene; (**c**) corresponding heights of traces 1, 2 and 3 with the heights in the range of 4.3–4.6 nm, adapted with permission from [68]. (**d**) Schematic diagram of CVD growth device; (**e**) AFM images of Te nanosheets; (**f**) the growth mechanism of 2D Te material at different temperatures, adapted with permission from [69]. (**g**) Large-area STM image of a Te film grown on a SiC substrate step (blue arrows); the inset shows the height line profile along the dark green line. (**h**) High-resolution STM image of a monolayer Te, revealing the rectangular lattice (inset arrows b and c); (**i**) differential STM images of Te islands on graphene/SiC, identifying layer increments from L1 to L4; (**j**) thickness-dependent evolution of the bandgap of Te films, adapted with permission from [70].

LPE is an efficient technique for preparing two-dimensional materials by weakening van der Waals interactions in bulk crystals and dispersing atomically thin layers into liquid media. Zhang et al. [68] used LPE to obtain tellurium nanosheets; the exfoliation process is shown in Figure 3a, and the thickness is confirmed to be 4.3–4.6 nm by the AFM measurements (Figure 3b,c). However, because LPE is sensitive to processing conditions, improper operation may result in significant differences in the thickness of the resulting two-dimensional nanosheets. Zhao et al. [69] later synthesized high-quality tellurene by CVD (Figure 3d), obtaining flakes with thicknesses of about 70 nm (Figure 3e). Their study showed that temperature plays a crucial role in determining morphology (Figure 3f). At low temperatures (<350 °C), Te atoms have limited mobility and preferentially grow along the intrinsic helical-chain direction, leading to one-dimensional nanowires. As temperatures increase to an intermediate range (350–380 °C), the atoms acquire enough energy to migrate more freely across the substrate, enabling lateral growth and leading to the formation of a 2D nanostructure. At temperatures exceeding 400 °C, isotropic growth dominates, resulting in spherical nanoparticles. Thus, increasing temperature drives the morphology evolution from 1D nanowires to 2D nanosheets and ultimately produces the spherical particles. In addition to solution-based synthesis, Huang et al. [70] reported vdW epitaxial growth of Te films on graphene by MBE, obtaining monolayer and few-layer 2D Te films (Figure 3g). Scanning tunneling microscopy revealed helically aligned Te chains distributed across the graphene surface. Figure 3h shows an STM image of a monolayer Te film, whereas Figure 3i,j demonstrate that the bandgap decreases monotonically with increasing film thickness. The bandgap value decreased monotonically with increasing thickness, from 0.92 of a monolayer to 0.49 of a 13-layer. In addition, the author employed ultrahigh-vacuum molecular beam epitaxy (UHV-MBE), combined with a co-evaporation strategy, by utilizing Sb_2_Te_3_ as a substrate and silicon as a “sacrifice agent”, achieving the epitaxial growth of monolayer α-tellurene [71]. In their study, the existence of silicon forms the a-Si:Te alloy and breaks the long Te chains, finally facilitating the formation of the short Te_3_ chains. The Te_3_ chains adsorbed onto the Sb_2_Te_3_ substrate and underwent a spontaneous structural phase transition to form monolayer α-tellurene.

Te semiconductor synthesis strategies mentioned above each have their unique advantages. For instance, the hydrothermal method offers the easy setup and advantage of large-scale production, while the vacuum phase deposition provides excellent crystallinity structure and high mobility. The crystallinity, scalability, advantages, and disadvantages of these synthesis strategies are summarized and compared in Table 1.

**Figure 4:**
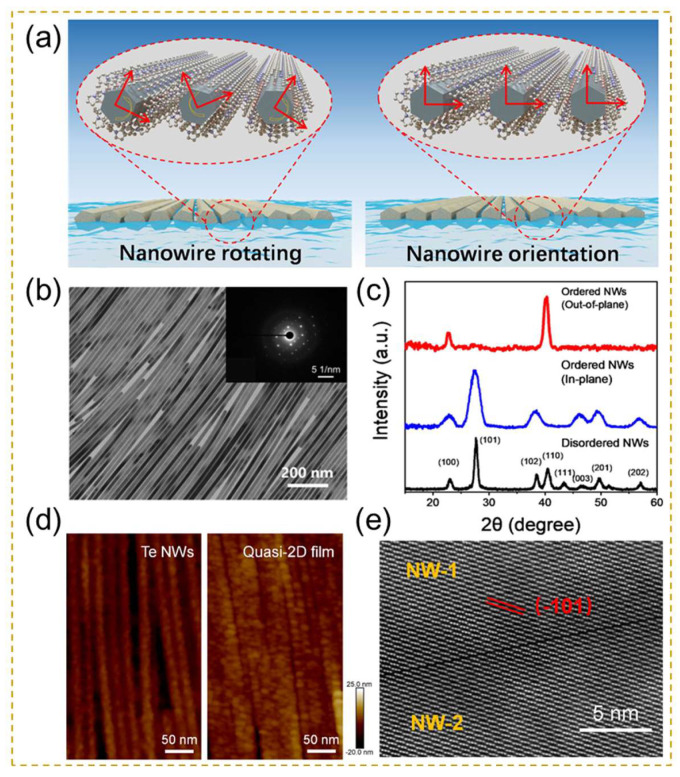
(**a**) Schematic diagram of TeNWs floating on water, with their crystal planes rotating from disordered to ordered. (**b**) TEM image of an ordered TeNW film; the inset shows the corresponding selected area electron diffraction (SAED) pattern confirming crystallinity. (**c**) X-ray diffraction (XRD) patterns of TeNW films with ordered (out-of-plane and in-plane) and disordered structures. (**d**) Height distribution diagrams of oriented TeNWs and quasi-two-dimensional nanofilms. (**e**) Scanning transmission electron microscopy (STEM) image highlighting the attachment interface of a quasi-2D nanofilm, adapted with permission from [33].

Besides the direct growth of Te nanostructures, Yu et al. [33] proposed an “interfacial-assembly-induced in situ synthesis” (IAIS) strategy to transform highly oriented one-dimensional TeNWs into centimeter-scale quasi-two-dimensional Te nanofilms. TeNWs with diameters of 12–19.5 nm were first synthesized hydrothermally and then assembled into an oriented nanowire film on the water surface by the Langmuir–Blodgett technique. As shown in Figure 4a, the TeNWs initially formed on the liquid–air interface with random crystal orientations. Then, an in situ IAIS strategy is applied at the liquid–air interface. They were then rotated to reduce the interaction energy to assemble TeNWs in an ordered orientation. The ordered nanowire film was then transferred onto ethylene glycol and heated at 180 °C under vacuum, which triggered oriented attachment of neighboring nanowires and their fusion into a continuous quasi-2D nanofilm. During heating, the PVP surfactant gradually dissolved into ethylene glycol, removing the steric barrier and weakening repulsive interactions between adjacent nanowires. As a result, attractive interactions between neighboring (100) facets became dominant and drove interwire attachment. The TEM image in Figure 4b reveals the ordered crystal planes, whereas the XRD patterns show that the ordered film exhibits fewer diffraction peaks than the disordered film and that the (110) is oriented perpendicular to the substrate (Figure 4c). In addition, the AFM height-mapping image of Figure 4d shows the surface fluctuations of the quasi-2D nanofilm, and the STEM image in Figure 4e confirms close attachment between adjacent TeNWs. In summary, the synthesis strategies of TeNWs and tellurene are listed in Table 2.

## 4. Applications

As a typical p-type semiconductor, Te has significant potential for the advancement of next-generation semiconductor electronics and optoelectronic applications. This section provides a comprehensive review of its emerging applications, specifically focusing on field-effect transistors, integrated logic gates, photodetectors, and the development of neuromorphic computing, including memristors and artificial synapse devices.

### 4.1. Field-Effect Transistors and Logic Gates

**Figure 5:**
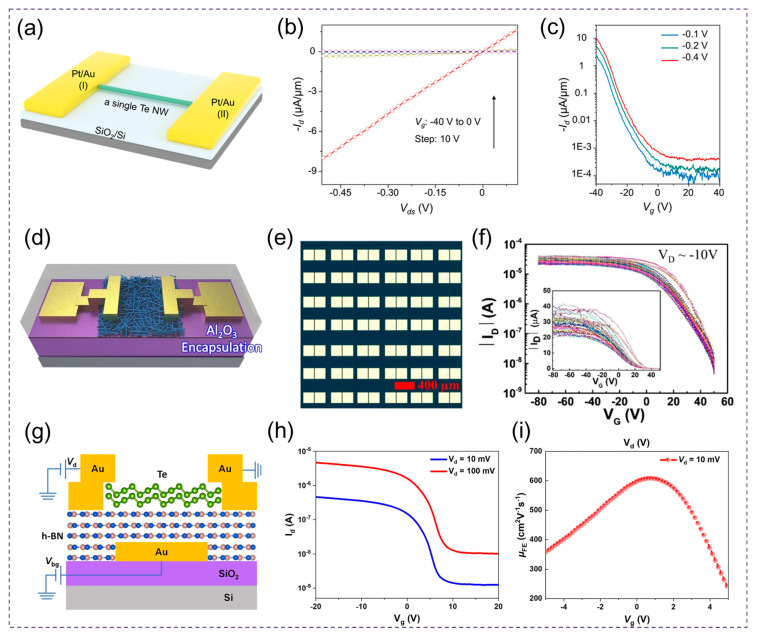
(**a**) Schematic structure of the single TeNW FET; (**b**) output characteristic of the single TeNW FET; (**c**) transfer characteristics of the single TeNW FET at different bias voltages, adapted with permission from [83]. (**d**) schematic illustration of the TeNW-network FET; (**e**) optical image of the TeNW-network FET array; (**f**) transfer curves of 42 devices on a log scale and linear scale (inset), adapted with permission from [31]; (**g**) schematic structure of the Te FET ultilizing h-BN as a dielectric layer; (**h**) transfer curves of the Te FET under different bias voltages; (**i**) field-effect mobility of Te FET extracted from the transfer curve at the bias voltage of 10 mV, adapted with permission from [84]. For (**b**), this is the output characteristics of the FET recorded from Vds ranging from −0.5 V to 0.5 V with Vg varied from −40 V to 0 V. For (**f**), this is the transfer characteristics of 42 devices. The author marked each transfer characteristic of the device in a different color to show the uniformity.

Recently, Guo et al. [83] developed a solution method to prepare FETs based on single TeNWs; the schematic structure is shown in Figure 5a. The single TeNW exhibits typical p-type behavior (Figure 5b), and the mobility can be extracted from the transfer curve in Figure 5c, demonstrating an excellent hole mobility of 417.8 cm^2^ V^−1^ s^−1^ and a current on/off ratio of 2.59 × 10^4^. The solution method has a significant advantage in fabricating large-scale electronics. Naqi et al. [31] developed large-scale FETs under a low-temperature process. The synthesized TeNW-network was uniformly coated onto the substrate using bar-coating followed by an etching process; the schematic structure is shown in Figure 5d. The proposed FET array containing 42 devices (Figure 5e) was measured and the transfer characteristics demonstrated 100% device yield and high uniformity, as shown in Figure 5f. The mobility is statistically 2.3–4.7 cm^2^ V^−1^ s^−1^ and the current on/off ratio is in the range of 10^3^–10^4^. In addition, Yang et al. [84] developed an ultrahigh-hole-mobility FET by growing tellurene on h-BN. The schematic architecture is shown in Figure 5g; the tellurene is grown on h-BN without any further transfer. The transfer curves were measured under bias voltages of 10 and 500 mV, respectively, and demonstrated p-type behavior and high crystal quality of tellurene, as shown in Figure 5h. Figure 5i is the mobility extracted from the transfer curve under the bias of 10 mV, extracting a peak mobility of 1370 cm^2^ V^−1^ s^−1^. Such high-mobility FETs have significant potential applications in integrated logic circuits.

**Figure 6:**
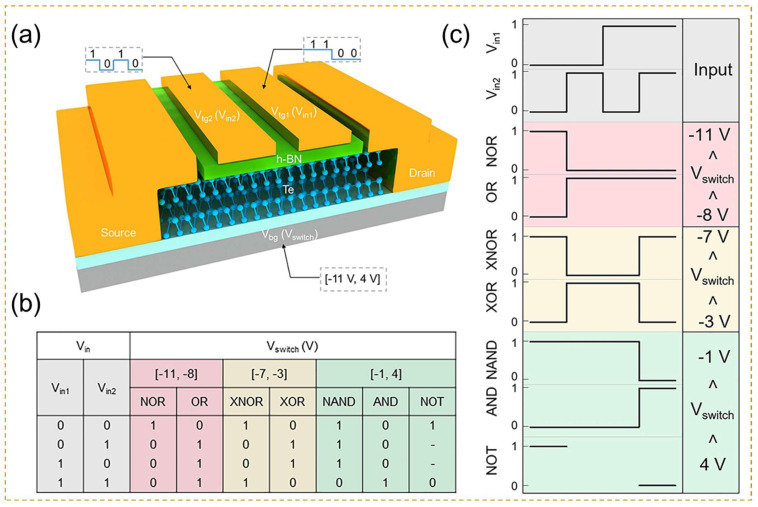
(**a**) Schematic diagram of the reconfigurable Te logic operator. (**b**) Comprehensive truth table for the Te logic operator. (**c**) Signal waveforms for seven logic gates (NOR, OR, XNOR, XOR, NAND, AND, and NOT) utilizing an ambipolar Te homojunction configuration, adapted with permission from [85].

In 2023, Zheng et al. [85] developed a reconfigurable logic operator based on a single ambipolar Te FET. The researchers employed an enhanced noninvasive scanning probe lithography (SPL) process together with a water-soluble PMMA/MA sacrificial layer, successfully yielding a bipolar Te FET suitable for advanced logic operations. Although the core device is a dual-gate Te homojunction that forms the basic p-n diode, the reconfigurable logic operator itself adopts a triple-gate architecture, with two independent top gates as logic inputs (V_in1_ and V_in2_) and a back gate V_bg_ serving as the switching gate (V_switch_); the schematic structure is shown in Figure 6a. By modulating the V_switch_ ranges, such as [−11 V, −8 V], [−7 V, −3 V] and [−1 V, 4 V]), together with suitable pull-up or pull-down resistor configurations, a single switchable Te FET can be engineered to perform all seven fundamental logic operations: AND, OR, XOR, NAND, NOT, NOR, and XNOR (Figure 6b,c). This single device with multifunctional integration significantly reduces circuit complexity and power consumption, making Te a key component for the next-generation energy-efficient programmable logic arrays and complementary metal-oxide-semiconductor (CMOS) technologies.

**Figure 7:**
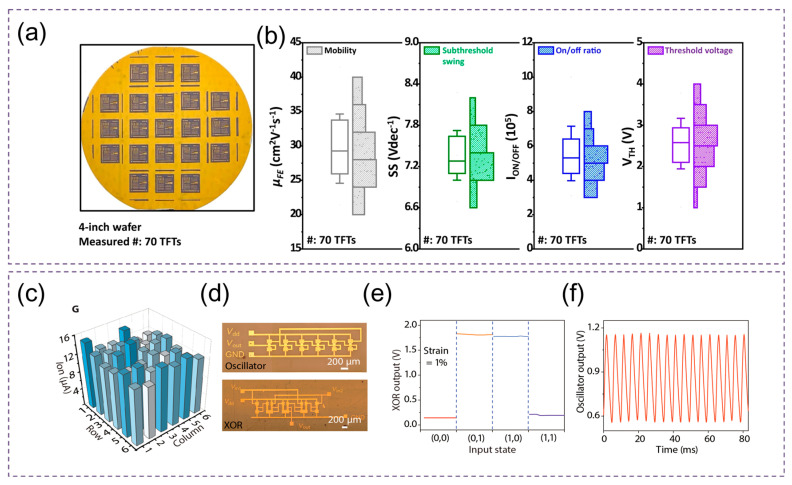
(**a**) Photograph of the Te FET array fabricated on a 4-inch Si/SiO_2_ (100 nm) wafer. (**b**) Statistical distribution of the mobility, subthreshold swing (SS), I_ON/OFF_ ratio and threshold voltage (V_TH_) across 70 individual FETs in the wafer-scale array—error bars indicate the standard deviation—adapted with permission from [40]. (**c**) On-state current (I_ON_) mapping for 6 × 6 array TeNW-FETs. (**d**) Optical micrographs of a ring oscillator and a XOR logic gate. (**e**) Output characteristics of the XOR logic gate under mechanical bending at V_DD_ = 2 V. (**f**) Output waveform of the five-stage ring oscillator operating at V_DD_ = 2 V, adapted with permission from [59].

Kim et al. [40] reported a low-temperature (150 °C) process for fabricating high-quality ultrathin crystalline Te films with a thickness of 4 nm and wafer-scale uniformity. The resulting FET arrays exhibited excellent electrical characteristics, and statistical evaluation of 70 devices confirmed their high uniformity and reproducibility (Figure 7a,b). Zhu et al. [59] also developed high-performance flexible and stretchable thin-film transistors and integrated circuits based on tellurium nanowires. By mimicking the hydrodynamic behavior of jellyfish tentacles, the authors investigated a “lock-and-shear” strategy to assemble highly oriented TeNWs on the substrate. When the nanowires with negative charges make contact with a positively charged substrate, the nanowires immediately adsorb and are fixed on the surface. These fixation points act as a “lock” for the orientation process, alongside the “shearing” from the water flow forces. The performance of TeNW-FETs achieved a maximum mobility of 116.1 cm^2^ V^−1^ s^−1^ and a current on/off ratio as high as 10^5^. They further integrated these materials into logic circuits. As illustrated in Figure 7c, the fabricated devices show a high on-state current and remarkable device uniformity. Besides the individual device, functional circuits including a ring oscillator and a XOR logic gate were also demonstrated, as shown in Figure 7d. Analyzing the two-bit input signals at a low driving voltage of 2 V demonstrated the clear and distinct output characteristics, as shown in Figure 7e. Furthermore, the ring oscillator in Figure 7f delivered a stable and continuous oscillation frequency of 200 Hz at V_dd_ = 2 V.

### 4.2. Photodetectors

**Figure 8:**
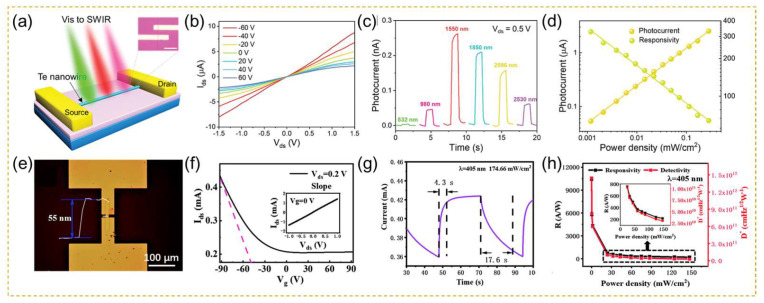
(**a**) Schematic illustration of the TeNW phototransistor. (**b**) I–V output characteristics of the phototransistor at different gate voltages. (**c**) The photoresponse of the phototransistor in a range of 532 nm to 2530 nm. (**d**) Photocurrent and responsivity versus power density, adapted with permission from [66]. (**e**) Optical image of the typical FET with a Te flake at 55 nm thickness. (**f**) Transfer characteristics of the typical FET; inset—output curve of the typical FET. (**g**) The photoresponse under 405 nm laser illumination. (**h**) The responsivity and specific detectivity are dependent on the power density; inset—enlarged part of the curve; adapted with permission from [86].

Because of their high carrier mobility and pronounced optoelectronic anisotropy, tellurium nanostructures are attractive candidates for high-performance photodetectors. Wei et al. [66] developed a TeNW-based phototransistor with a broadband photoresponse spanning 532 to 2530 nm. A back-gate FET architecture was used for electrical and photoelectrical measurements (Figure 8a). The linear current–voltage characteristics indicated ohmic contact between the TeNWs and the electrodes (Figure 8b). Figure 8c shows the photoresponse under laser illumination at 532, 980, 1550, 1850, 2096, and 2530 nm, with the highest response observed at 1550 nm. Under 1550 nm illumination with a power density of 0.0012 mW cm^−2^, the responsivity reached 327 A W^−1^ (Figure 8d). Huang et al. [86] also fabricated photodetectors based on vapor-deposited 2D Te flakes. Figure 8e shows an optical image of a device with a Te flake thickness of 55 nm, and the transfer and output characteristics measured in the dark are presented in Figure 8f. Under 405 nm illumination, the rise and decay times were 4.3 and 17.6s, respectively. Moreover, the device exhibited an ultrahigh photoresponsivity of 1.04 × 10^4^ A W^−1^ and a specific detectivity of 1.4 × 10^12^ Jones, as shown in Figure 8g,h.

**Figure 9:**
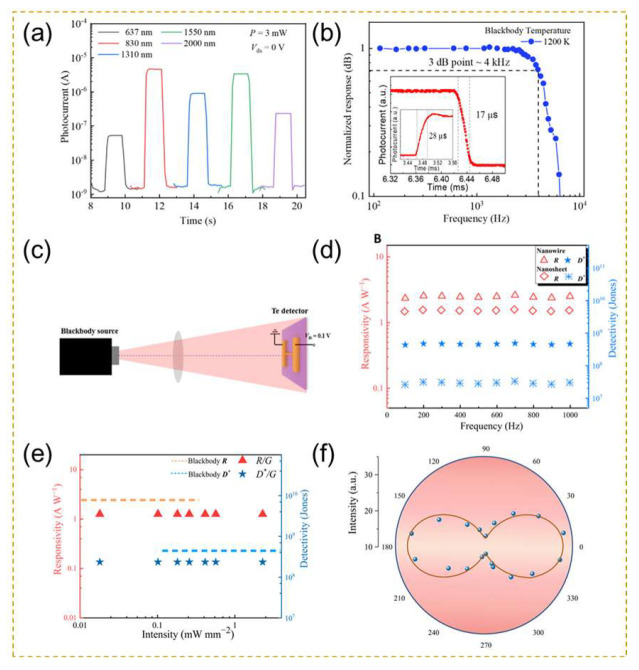
(**a**) Photocurrent dependent on time of the photodetector under different wavelength illumination at 3 mW power. (**b**) Normalized photoresponse versus modulated frequency under blackbody illumination at 1200 K; inset—rise time and decay time are 28 μs and 17 μs, respectively; adapted with permission from [87]. (**c**) Schematic diagram of the photodetector under the illumination of blackbody source. (**d**) Blackbody responsivity and detectivity performance vary with the frequency. (**e**) Pure responsivity and detectivity dependent on the intensity under 1550 nm light illumination. (**f**) Polarization properties of Te photodetector, adapted with permission from [88].

Blackbody response is a standard metric for infrared focal-plane-array detectors and reflects the sensitivity. Peng et al. [87] reported a blackbody-sensitive photodetector based on a Te/graphene heterojunction. Figure 9a shows the photoresponse over a wide spectral range from 637 to 2000 nm. The device shows a clear photoresponse under 1200 K blackbody radiation, and the 3 dB bandwidth, defined as the frequency at which the responsivity drops to 0.707 of its low-frequency value, reached 4 kHz, as shown in Figure 9b. In addition, a peak detectivity of 3.69 × 10^8^ cm Hz^1/2^ W^−1^ was achieved under 1200 K blackbody radiation. Furthermore, the authors also developed an infrared photodetector based on tellurium nanowires and nanosheets [88]. Figure 9c shows the schematic blackbody detection setup. Using a 1200 K blackbody source, the responsivity and detectivity reached 2.53 A W^−1^ and 4.68 × 10^8^ Jones, respectively, and remained stable at 1000 Hz, as shown in Figure 9d. Under 1550 nm illumination, the device exhibited a peak responsivity of 5.19 A W^−1^ and a detectivity of 9.6 × 10^8^ Jones, indicating gain-assisted blackbody detection, as shown in Figure 9e. Figure 9f further shows that the Te photodetector possesses good polarization-sensitive photoresponse.

**Figure 10:**
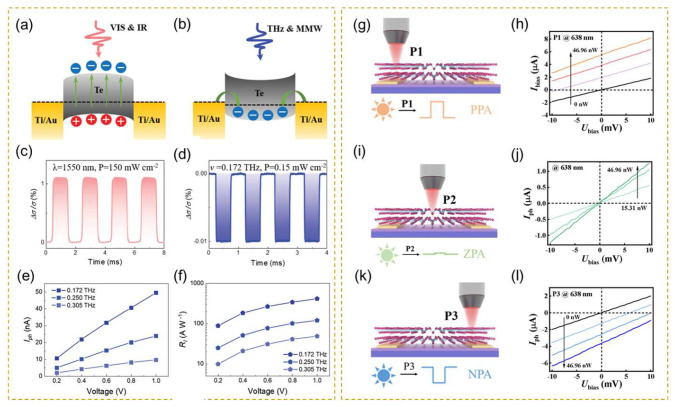
(**a,b**) Schematic diagram of the device under (**a**) VIS and IR and (**b**) THz and MMW illumination, (**c**) positive photoconductance under 1550 nm light illumination at 150 mW cm^−2^ power density, and (**d**) negative photoconductance on 0.172 THz illumination at 0.15 mW cm^−2^. The photocurrent (**e**) and responsivity (**f**) dependent on the voltage bias were measured from 0.2 to 1.0 V at 0.172, 0.250, and 0.305 THz. Image adapted with permission from [89]. (**g**,**i**,**k**) Schematic illustration of the photodetector under localized illumination at P1, P2 and P3, respectively. (**h**,**j**,**l**) The I-V curve was measured in dark conditions and varying light illuminations. Image adapted with permission from [90].

Ma et al. [89] demonstrated an ultrawideband photodetector utilizing elemental Te with a detection range spanning the visible, infrared, terahertz (THz), and millimeter-wave (MMW) regions. A key finding of this work is that the response arises from the combined contributions of photogenerated electron–hole pairs and the electromagnetic-induced well effect (Figure 10a,b). The responsivities were 0.793 A W^−1^ and 9.38 A W^−1^ under 635 nm and 1550 nm illuminations, respectively. In the THz and MMW regimes, the responsivities reached 9.83 A W^−1^, 24.8 A W^−1^, and 87.8 A W^−1^ at 0.305, 0.250 and 0.172 THz, respectively (Figure 10c–f), highlighting the excellent performance of Te in long-wavelength photodetection.

Researchers have also explored how to actively switch and balance two fundamental photocarrier-transport mechanisms in a single Te optoelectronic device: the drift-driven photoconductive effect and the diffusion-driven photothermoelectric effect. Ni et al. [90] designed a photodetector based on suspended TeNWs that exhibited three programmable photocurrent states: positive photocurrent (PPA), zero photocurrent, and negative photocurrent (NPA) (Figure 10g,i,k). The current–voltage behavior confirmed ohmic contact between the TeNWs and the electrodes, indicating drift-dominated transport in the P1 and P3 states, whereas diffusion transport dominated in the P2 state, as shown in Figure 10h,j,l. Huang et al. [91] also reported a cost-effective near-infrared photodetector based on a self-assembled TeNW film/Si heterostructure. This type II band-aligned heterostructure effectively promotes the separation and transmission of photogenerated carriers, thereby significantly reducing dark current and improving photoelectric conversion efficiency. Under 808 nm near-infrared illumination, the detector achieved a responsivity of 0.12 A/W and a high detectivity of 3.5 × 10^10^ Jones.

Additionally, representative Te-based photodetectors are summarized in Table 3. Their response range extends from the UV to millimeter-wave regions, highlighting the broad potential of tellurium nanostructures for broadband photodetection.

### 4.3. Memristor Device

**Figure 11:**
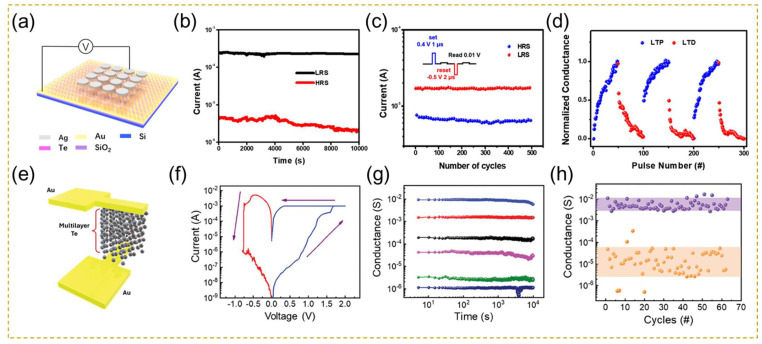
(**a**) Schematic structure of the fabricated Te vertical memristor; (**b**) retention characteristics of high-resistance states (HRS) and low-resistance states (LRS); (**c**) endurance testing of the device; (**d**) continuous testing of long-term potentiation (LTP) and long-term depression (LTD), adapted with permission from [108]; (**e**) schematic of Au/Te/Au memristor; (**f**) I–V curve of the device shows resistive switching; (**g**) 6-level state retention characteristics for long-term memory analysis; (**h**) DC switching endurance test over 60 cycles, adapted with permission from [109].

Li et al. [108] developed a vertical memristor utilizing a large-area 2D Te film, and the schematic device structure is shown in Figure 11a. The retention data indicate a high-to-low resistance ratio greater than 10, with both states maintained for up to 10,000 s, as shown in Figure 11b. Then, the endurance test further showed that the device could be rewritten more than 500 times, demonstrating good durability, as shown in Figure 11c. Moreover, the memristor was used to emulate synaptic behavior under continuously applied voltage pulses. In Figure 11d, the conductance gradually increased from 12 to 19 μS over 50 consecutive pulses with an amplitude of 0.4 V and a width of 5 μs, then decreased from 19 to 12 μS over 50 consecutive pulses of −0.5 V and 10 μs, corresponding to potentiation and depression, respectively. Ghomi et al. [109] also demonstrated that direct growth of Te films on Au substrates can reduce the operating voltage and energy consumption of memristive devices. The device structure is shown in Figure 11e. Bipolar voltage sweeps yielded a maximum on/off ratio of 1.37 × 10^5^ (Figure 11f). Conductance states ranging from 1 µS (HRS) to 10 mS (LRS) exhibited retention for more than 10^4^ s across six programmable levels (Figure 11g), and the endurance test showed stable operation for more than 60 cycles, as shown in Figure 11h. In addition, Zhang et al. [110] reported vertical ferroelectric behavior in TeNWs, which enabled the fabrication of a self-gated ferroelectric field-effect transistor with excellent nonvolatile resistive switching and multilevel resistance states.

### 4.4. Artificial Synapses

**Figure 12:**
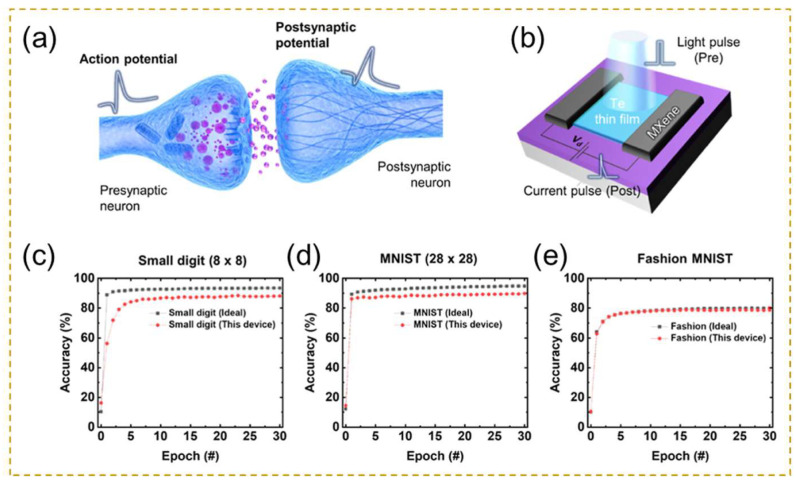
(**a**) Schematic illustration of a biological synapse; (**b**) working principle of the Te photonic synapse; (**c**–**e**) recognition accuracy plots of the simulated neural network for different datasets—(**c**) small digit, (**d**) MNIST, and (**e**) Fashion MNIST—adapted with permission from [112].

Bian et al. [111] reported a 2D Te-based threshold-switching memristor with low variation in the high-resistance state, which was further used to emulate an artificial nociceptor—an electronic device that mimics the human hand retraction reflex. The working principle shows that if the input voltage exceeds the threshold, the memristor switches from the HRS to the LRS, corresponding to a painful external stimulus. Conversely, the device remains in the HRS if the voltage is below the threshold voltages, indicating a harmless stimulus. In addition, Jo et al. [112] introduced an artificial photonic synapse based on a Te thin film with MXene electrodes. The biological synapse model and the operating mechanism of the Te photonic synapse are illustrated in Figure 12a,b. In neural systems, an action potential arriving at the presynaptic neuron triggers neurotransmitter release. These chemicals are captured by the dendrites of the postsynaptic neuron, and cause membrane depolarization. When depolarization exceeds a threshold, an excitatory postsynaptic potential is generated, enabling signal transmission across synapses. In the artificial device, a presynaptic light pulse induces a source–drain current known as the excitatory postsynaptic current, which is analogous to the biological response. This device was further evaluated in neural-network simulations, where the recognition accuracy for the small MNIST and MNIST datasets approached 90%, only about 5% lower than the ideal case. For the Fashion-MNIST dataset, the simulated and ideal networks showed similar accuracies (Figure 12c–e).

**Figure 13:**
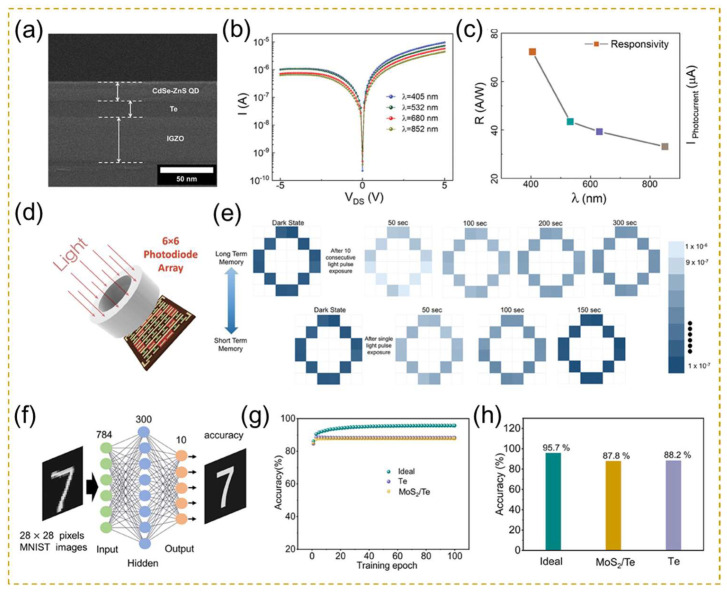
(**a**) Cross-sectional SEM micrograph of IGZO-Te-QD heterostructure; (**b**) optical response of the device for different light sources from Visible to NIR range; (**c**) responsivity of the device for different excitation source energy (wavelength); (**d**) 3D layout of the fabricated 6 × 6 photodiode array; (**e**) mapping representation of the fabricated 6 × 6 photodiode array for demonstrating short-term and long-term memory, adapted with permission from [114]; (**f**) architecture of an artificial neural network configured for the recognition of 28 × 28 pixel image of the MNIST handwritten digit “7”; (**g**) comparative analysis of image recognition accuracy with 100 epochs in the ideal simulation, Te and MoS_2_/Te device; (**h**) final recognition accuracy of ideal, Te and MoS_2_/Te networks after 100 epochs, adapted with permission from [115].

Introducing Te into the heterojunction devices can effectively extend the spectral response into the visible and even infrared regions. Recently, Pan et al. [113] reported a Te/GaN hybrid heterojunction photodetector with a broadband photoresponse from 200 to 2500 nm and synaptic-like functions. The key innovation lies in exploiting the spontaneous polarization of the wide-bandgap GaN layer to induce a functional charged Te interfacial layer. Dutta et al. [114] designed a photodiode based on a quantum-dot-enhanced IGZO/Te heterostructure to emulate key functions of biological synapses. Quantum dots (QDs) are widely used in photodetectors because of their tunable bandgaps and broad spectral absorption, whereas IGZO is attractive for its high on/off ratio and high electron mobility. Therefore, their combination extends the photoresponse into the near-infrared region. The cross-sectional SEM image shows QD, Te, and IGZO layer thicknesses of 16, 11, and 45 nm, respectively, as shown in Figure 13a. The current–voltage curves measured under different wavelengths reveal a broadband response range from visible to the near-infrared wavelengths, as shown in Figure 13b, and the corresponding responsivities are shown in Figure 13c. Furthermore, image-memory experiments were carried out using a 6 × 6 photodiode array (Figure 13d). The results showed that the array retained an “O” pattern for 150 s in short-term memory mode and for 300 s in long-term memory mode, directly demonstrating its capabilities for image perception, processing, and memory, as shown in Figure 13e.

Li et al. [115] introduced a vdW p-n heterojunction memristor based on MoS_2_ and tellurene. Memristors are considered ideal building blocks for neuromorphic computing due to their ability to integrate storage and processing functions while mimicking biological synaptic behavior. This specific device successfully reproduced a variety of key synaptic responses, including the transition from STP to LTP and PPF, and showed practical functionality as a logic element and in image recognition using an ANN. Furthermore, the researchers constructed a three-layer ANN containing 784 input neurons, 300 hidden neurons, and 10 output neurons by integrating light-enhanced and electrically inhibited conductance data, as shown in Figure 13f. Following 100 training epochs, the network based on the experimental device achieved a recognition accuracy of 87.8% (Figure 13g), which approached the simulated performance of an ideal device (95.7%, Figure 13h). Beyond rigid silicon-based platforms, flexible neuromorphic electronics are also in high demand. You et al. [116] therefore developed a flexible artificial synaptic transistor based on two-dimensional tellurene on a PET substrate. The energy consumption per pulse was only 9 fJ, comparable to the approximately 10 fJ consumed by a biological synapse in the human brain. The device also exhibited excellent synaptic plasticity, high linearity and symmetry, and up to 93 effective conductance states. Using a two-layer perceptron neural network, the authors simulated learning and recognition under different bending conditions. The MNIST handwritten-digit dataset was used to test the accuracy of the system; the recognition accuracy reached 93% in the flat state, 94% under convex bending, and 88% under concave bending. These results indicate that the device maintains high recognition accuracy even with mechanical bending, suggesting that it is suitable for integration into next-generation flexible and neuromorphic systems.

## 5. Conclusions and Perspectives

Te nanostructures, as natural p-type vdW semiconductors with a unique crystal structure and excellent carrier mobility, have been investigated as promising materials in various applications. In this review, we summarized the synthesis strategies of Te semiconductors, including the TeNWs and tellurene, and their recent emerging applications in electronics and optoelectronics. In the direct-synthesis strategies, the representative methods include hydrothermal growth, liquid-phase exfoliation, CVD, PVD and MBE, as well as the assembly methods from 1D TeNWs to 2D thin films, such as Langmuir–Blodgett assembly, blade coating, and lock-and-shear alignment methods.

Despite these advancements in synthesis strategies, several significant challenges remain, including long-term ambient stability of TeNWs, scalable synthesis of high-quality and high-uniformity tellurene, and CMOS-compatible integration of Te into complex circuits. The instability in the ambient is a major obstacle to the commercialization of Te devices. As a chalcogenide element, Te easily oxidizes on its surface when exposed to oxygen and moisture, resulting in the formation of tellurium oxides. These oxide phases introduce electrically active trap states and scattering centers, causing a decline in device performance over time. To address this issue, various surface passivation and encapsulation strategies have been developed to protect Te from oxidation while enhancing device electrical performance. The strategies can be categorized into three types: inorganic encapsulation, organic–inorganic hybrid encapsulation, and van der Waals (vdW) material encapsulation. Looking forward, one promising approach is to first stack a polymer layer as a contact engineer to optimize electrical performance, then deposit a high-quality atomic layer (e.g., Al_2_O_3_) to achieve sealing against oxygen and moisture. Furthermore, the vdW material encapsulation is a cutting-edge method to achieve devices with high mobility, even though wafer-scale integration with Te remains a challenge. Therefore, by employing customized synergistic passivation methods, the inherent instability of Te can be eliminated, thus paving the way for its successful commercialization in next-generation electronics and optoelectronic devices. In addition, a major bottleneck of tellurene preparation is the lack of a scalable synthesis method for fabricating large-scale uniform, single-crystal tellurene films. While the hydrothermal method is cost-effective and high-throughput, the production usually small and randomly oriented nanosheets, limiting carrier mobility. The vacuum vapor deposition can achieve high-crystallinity and exceptional mobilities; however, the film uniformity and thickness control growth on the wafer remains a challenge. Therefore, the challenge is to develop a technique that enables the realization of low-cost, high-throughput, and high-crystallinity tellurene fabrication on a large scale.

Te semiconductor devices in field-effect transistors, photodetectors, and logic gates demonstrate the material potential to extend Moore’s law. Specifically, the Te narrow bandgap promotes high photoresponse across the UV to mid-wave infrared regions, making it an ideal platform for next-generation photodetectors. In addition, the emergence of Te-based memristors and synaptic devices demonstrates great potential for application in neuromorphic computing and in-sensor processing. With continued advances in device engineering and device architecture design technologies, Te is expected to play an increasingly significant role in the future of advanced semiconductor technologies.

## Figures and Tables

**Table 1:** Summary and comparison of the Te synthesis strategies.

Synthesis Strategy	Scalability	Crystallinity	Advantages	Disadvantages/Challenges
Hydrothermal	High	Single-crystalline	Cost-effective and easy setup	Use toxic reducing agents and high pressure
			High throughput and large-scale production	Surfactant residues easily cause chemical contamination
			Controllable morphologies	Lower crystallinity
				Higher defect density and limited carrier mobility
CVD/CVT	Medium	Single-crystalline	High-quality, single-crystallinity	High-cost and complex equipment
			Very high carrier mobility	High growth temperatures and specific substrates
			Direct growth in a gas environment without solvent defects	Difficult to maintain thickness uniformity in large area
PVD	Medium	Single-crystalline	High crystallinity and quality	High-cost
			Good controllability over film thickness and orientation	Requires high-purity sources and vacuum environment
			Suitable for 1D/2D heterojunctions	
TE	High	Polycrystalline	Large-area deposition	
			Arbitrary substrates	Low film density
			Low-temperature process	Less thickness uniformity that produces polycrystalline films
			Extremely high deposition rate	
MBE	Low	Single-crystalline	Atomic-level precision thickness control	Very high cost and complexity
			High purity and cleanliness	Extremely low growth
			Suitable for producing high-quality, 2D single-crystalline films	Difficulty in large-scale mass production
LPE	High	Single-crystalline	High efficiency for producing 2D Te layers (tellurene)	Easily introduces lattice defects
			Low-cost, highly processable solution	Uneven size distribution and random thickness
			Large-scale production	Not suitable for large-area, continuous thin films
			Can be integrated with printing methods	

**Table 2:** Summary of the synthesis strategies of TeNWs and tellurene.

Synthesis Strategy	Te Source	Reductant	Surfactant	Reaction Media	Solvent	Morphology	Ref.
LPE	Te powder	—	—	Ultrasonication (400 W)	IPA	nanosheets	[12]
Magnetron sputtering	Te	—	—	2 mTorr (20 W)	—	nanosheets	[40]
Hydrothermal	Na_2_TeO_3_	—	—	HCl	Ethanol & DI water	nanowires	[51]
One-pot synthesis	TeO_2_	EG	PVP	NaOH	EG	nanowires	[55]
CVD	Te powder	—	—	vacuum	—	nanosheets	[69]
Hydrothermal	Te powder	N_2_H_4_	—	—	DI water	nanowires	[72]
Hydrothermal	Na_2_TeO_3_	Glucose	CTAB	—	DI water	nanowires	[73]
Hydrothermal	Na_2_TeO_3_	N_2_H_4_	PVP	NH_3_·H_2_O	DI water	nanowires	[74]
Hydrothermal	Na_2_TeO_3_	Sucrose	—	—	DI water	nanowires	[75]
One-pot synthesis	Na_2_TeO_3_	Ascorbic acid	—	KOH	EG & DI water	nanowires	[76]
PVD	Te powder	—	—	Ar gas	—	nanowires	[77]
One-pot synthesis	NaHTe		PVP	—	ethanol	nanosheets	[78]
PVD	Te powder	—	—	Vacuum	—	nanosheets	[79]
Hydrothermal	Na_2_TeO_3_	N_2_H_4_	PVP	NH_3_·H_2_O	DI water	nanosheets	[80]
LPE	1T’-MoTe_2_	—	—	Ultrasonication (140 W)	NMP	nanosheets	[81]
Thermal evaporation	Te pellets	—	—	Vacuum (14 W)	—	nanosheets	[82]

**Table 3:** Summary of the Te-based photodetectors.

Materials	Response Spectrum	Responsivity	Detectivity (Jones)	EQE	Ref.
Te	520 nm–3.39 μm	383 A W^−1^	1.9 × 10^3^	—	[14]
Te/Ge	465–980 nm	523 mA W^−1^	9.50 × 10^10^	—	[23]
Te/Si	450–1870 nm	437.24 A W^−1^	4.86 × 10^11^	—	[36]
Te	450 nm–10.6 μm	4.69 A W^−1^	1.48 × 10^11^	—	[92]
Te	1550 nm	51.85 A W^−1^	1.88 × 10^10^	4148%	[93]
Te/Si	1300 nm	248 mA W^−1^	1.8 × 10^12^	91%	[94]
Te	365–1310 nm	1189 A W^−1^	1.15 × 10^9^	—	[95]
Te	1550 nm	26.1 A W^−1^	3.24 × 10^9^	2090.9%	[96]
Te/MoS_2_	520–1550 nm	1.51 A W^−1^	2.55 × 10^10^	360.77%	[97]
Te/Bi_2_Te_3_	365–850 nm	12 mA W^−1^	5.87 × 10^10^	41.05%	[98]
Te_0.65_Se_0.35_ alloy	405–1550 nm	7.35 A W^−1^	1.32 × 10^9^	1440%	[99]
Te	408 nm	72.94 A W^−1^	2.23 × 10^10^	—	[100]
Te/Bi_2_Se_3_	365 nm–4.3 µm	0.88 A W^−1^	1.77 × 10^10^	—	[101]
TeNW/WS_2_	635 nm	0.471 A W^−1^	1.24 × 10^12^	91%	[102]
Te/ReS_2_	632 nm	180 A W^−1^	7.2 × 10^9^	—	[103]
Te/TiO_2_	300–500 nm	0.387 A W^−1^	4 × 10^10^	—	[104]
Te/MoTe_2_	520–1310 nm	30.1 A W^−1^	4.9 × 10^11^	7.16 × 10^3^%	[105]
Te/MoS_2_	980 nm–3.0 μm	28.4 A W^−1^	2.7 × 10^10^	5.7 × 10^3^%	[106]
Te/CsPbBr_3_	300–500 nm	0.35 mA W^−1^	1.42 × 10^10^	—	[107]

## Data Availability

No new data were created or analyzed in this study.

## Abbreviations

The following abbreviations are used in this manuscript:

TeNWs	Tellurium nanowires
FET	Field-effect transistors
TEM	Transmission electron microscope
AFM	Atomic force microscopy
STEM	Scanning transmission electron microscope
h-BN	hexagonal boron nitride
PMMA/MA	Poly(methyl methacrylate-co-methyl acrylate)
MNIST	Modified National Institute of Standards and Technology
IGZO	Indium gallium zinc oxide
ANN	Artificial neural network
PET	Polyethylene terephthalate
